# A randomised controlled unblinded multicentre non-inferiority trial with activated vitamin D and prednisolone treatment in patients with minimal change nephropathy (ADAPTinMCN)

**DOI:** 10.1186/s13063-021-05393-4

**Published:** 2021-07-12

**Authors:** Tilde Kristensen, Henrik Birn, Per Ivarsen

**Affiliations:** 1 Department of Internal Medicine, renal unit, Regional Hospital Viborg, and Department of Clinical Medicine, Aarhus University, Aarhus, Denmark; 2 Department of Nephrology, Aarhus University Hospital and Department of Biomedicine, Aarhus University, Aarhus, Denmark; 3 Department of Nephrology, Aarhus University Hospital, and Department of Clinical Medicine, Aarhus University, Aarhus, Denmark

**Keywords:** Minimal change nephropathy, Prednisolone, Activated vitamin D, Nephrotic syndrome, Adverse events, Pharmacokinetics

## Abstract

**Background:**

Minimal change nephropathy (MCN) is a common cause of nephrotic syndrome in both adults and children. International guidelines recommend treatment with prednisolone 1 mg/kg/day to adults. This dose is derived from an empirically established dose in children, although children generally attain remission faster and relapse more rapidly than adults. Prednisolone is associated with multiple and serious adverse events. Activated vitamin D has been shown to reduce albuminuria in other glomerular renal diseases with a minimum of adverse events. This study tests the hypothesis that a new treatment regimen in MCN combining reduced dose prednisolone and active vitamin D is as efficient in inducing remission and has fewer and less severe adverse events than standard prednisolone. Furthermore, we aim to establish models allowing for more personalized medicine based on assessment of the individual’s prednisolone metabolism.

**Methods:**

A randomised controlled multicentre non-inferior unblinded trial including 96 adult, incident patients with biopsy-proven MCN, albuminuria > 3 g/day, and an estimated glomerular filtration rate (eGFR) > 30 ml/min from renal departments in Denmark. Patients are randomised to standard prednisolone (1 mg/kg/day) or reduced prednisolone (0.5 mg/kg/day) and alfacalcidol (0.5 μg/day). The primary outcome is the rate of remissions after 16 weeks and the time from diagnosis to remission. The study will include a saliva test to characterise prednisolone pharmacokinetics and compare them to genetic variations in specific liver enzymes responsible for prednisolone metabolism.

**Discussion:**

Reducing the prednisolone dose is expected to reduce the number of severe adverse events. This study will examine if reduced prednisolone dose with active vitamin D but without additional immunosuppression is feasible in the treatment of MCN and will reduce the number of adverse events. The findings can potentially change current guidelines for treatment of MCN in adults. Additional outcomes on inter-individual pharmacokinetic and metabolic variations may allow for a more personalised treatment strategy.

**Trial registration:**

EudraCT 2017-001206-16, ClinicalTrials.gov NCT03210688. Registered on June 3, 2017.

## Administrative information

The order of the items has been modified to group similar items (see http://www.equator-network.org/reporting-guidelines/spirit-2013-statement-defining-standard-protocol-items-for-clinical-trials/).

**Table:** 

Title {1}	A randomized controlled unblinded multicenter non-inferiority trial with **A**ctivated vitamin **D a**nd **P**rednisolone **t**reatment in patients with **m**inimal **c**hange **n**ephropathy (ADAPTinMCN)
Trial registration {2a and 2b}.	EudraCT: 2017-001206-16 Clinical trials: NCT03210688
Protocol version {3}	Protocol version 10.01.2020
Funding {4}	Financial fundings: The Augustinus Foundation, Aase and Ejnar Danielsens Foundation, Danish Society of Nephrology, Helen and Ejnar Bjørnow foundation, Nyreforeningen, Regional Hospital Central Jutland Viborg, The Health Research Fund of Central Denmark Region, and Aarhus University.
Author details {5a}	Tilde Kristensen, MD, Staff specialist in Nephrology, Department of Internal Medicine, renal unit, Regional Hospital Viborg, and Department of Clinical Medicine, Aarhus University, Aarhus, Denmark. Henrik Birn, DMSc, MD, Professor and consultant, Nephrologist, Department of Nephrology, Aarhus University Hospital and Department of Biomedicine, Aarhus University, Aarhus, Denmark. Per Ivarsen, PhD, MD, Consultant, Nephrologist, Department of Nephrology, Aarhus University Hospital, and Department of Clinical Medicine, Aarhus University, Aarhus, Denmark.
Name and contact information for the trial sponsor {5b}	Per Ivarsen, Ph.D., MD, Consultant, Nephrologist, Department of Nephrology, Aarhus University Hospital, Palle Juul-Jensens Boulevard 99, 8200 Aarhus N, Denmark. Mail: perivars@rm.dk
Role of sponsor {5c}	Study design, management, collection, analysis, interpretation of data, writing the report and decision to submit the report for publication.

## Introduction

### Background and rationale {6a}

Minimal change nephropathy (MCN) is one of the most common causes of nephrotic syndrome worldwide, and it is the underlying pathology in 10–25% of all cases in adults [1]. The nephrotic syndrome in MCN is characterised by an approximately 100-fold or higher increase in urinary albumin excretion, hypoalbuminemia, oedema, and significantly increased plasma lipids [2, 3]. The pathophysiology of MCN is not fully clarified but may be associated with a dysfunctional interaction between the immune system and the glomerular podocyte, which is essential for maintaining the glomerular filtration barrier. Most studies indicate that T cells are involved, although the effect of CD20 receptor antibody treatment indicates that the B cells also play a role.

MCN only rarely leads to end-stage renal disease. However, studies have shown that MCN is associated with an increased risk of mortality [4], cancer [5], and cardio-vascular events, including thromboembolic disease in both children and adults [6, 7]. It is unknown whether it is the disease or the associated treatment that induces long-term complications.

Currently, the Kidney Disease: Improving Global Outcomes (KDIGO) guidelines recommend a high dose of oral prednisolone (1 mg/kg/day) [8] for treatment of adult patients with MCN for up to 16 weeks, depending on their clinical response [8]. This dose is based on an empirical dose (60 mg/m^2^/day) suggested from studies in children [9]. Children, however, generally attain remission faster than adults with 50% being in remission within 8 days of treatment compared with 2 months to remission in adults [2]. Relapse occurs more rapidly in children than in adults and up to 90% of all children with MCN have at least one relapse [2]. In adults, relapse is seen in 30–70% of cases during tapering or after discontinuation of prednisolone [10–12]. According to guidelines, relapse should be treated with a new course of high-dose prednisolone. Thus, a great number of patients are exposed to a large, cumulative dose of prednisolone.

An older observational study in adult patients with nephrotic syndrome suggested that the time to remission is substantially shorter in patients treated with prednisolone than in untreated patients [13]. A small and underpowered study indicated that in adult MCN remission may be induced just as fast when using a smaller cumulative dose of prednisolone [14], than recommended in the present international guidelines, and with no significant differences in the cumulative numbers of relapse although a trend towards earlier relapse with the low-dose regimen was observed [14]. A systemic review comparing standard high-dose prednisolone to a low dose of prednisolone combined with other immunosuppressants suggested no difference in relapse rates [15].

Treatment with active vitamin D reduces proteinuria in various types of kidney disease [16–19]. Studies in patients with IgA nephropathy [16, 18, 20, 21] show that pharmacological doses of active vitamin D reduce urinary albumin excretion. Thus, clinical observations suggest that treatment with active vitamin D may stabilise the glomerular filtration barrier and thereby reduce albuminuria and provide additional benefit to prednisolone. However, this has never been tested in MCN. Furthermore, patients with nephrotic syndrome have low levels of vitamin D because of increased urinary excretion of vitamin-D-binding protein [22, 23], indicating possibly additional benefits from such treatment.

Treatment with prednisolone is associated with important physical and mental adverse effects, including an increased risk of infections, obesity, diabetes, and hypertension and mental changes, e.g. mood changes, depression, and agitation [24], which collectively constitute a strong argument for reducing the cumulative prednisolone dose. Recently, a Glucocorticoid Toxicity Index [25] has been developed to identify and quantitate prednisolone-related complications. The Index includes changes in body mass index, glucose tolerance, blood pressure, lipid status, bone density, steroid myopathy, skin toxicity, neuropsychiatric toxicity, infections, and adrenal insufficiency, as well as gastrointestinal and ocular disease. The Cushing Quality of life (QoL) questionnaire addresses known problem areas in Cushing and is useful for evaluating patient-reported health-related quality of life and correlates with clinical parameters [26, 27]. Also, the short form (SF) 36 is a well-established patient-reported survey that has been used to determine the health-related QoL in the general population and among different groups of patients, e.g. patients with renal disease [28].

The pharmacokinetics of prednisolone are complex. Prednisolone is protein bound, and its metabolism is influenced by gender and plasma albumin as well as by liver function and genetic variations in the enzymes CYP3A4, CYP3A5, ABCB1, and NR112 [29]. Repetitive samplings of non-stimulated saliva provide a good estimate of prednisolone turnover in healthy people [29] and in children with nephrotic syndrome [30]. This may allow for individualised dosing of prednisolone to reduce adverse events without compromising efficacy.

The aim of this study is to test the hypothesis that a new treatment regimen in MCN combining reduced dose prednisolone and active vitamin D is as efficient in inducing remission and has fewer and less severe adverse events than standard prednisolone. Furthermore, we aim to establish models allowing for more personalized medicine based on assessment of the individual’s prednisolone metabolism.

### Objectives {7}

Based on the potential benefits from reducing cumulative prednisolone dose and the possible positive effects of active vitamin D in nephrotic syndrome, the study will test the following hypothesis:

1. Treatment of MCN with a reduced dose of prednisolone in combination with active vitamin D is non-inferior to standard dose of prednisolone with respect to rate and time to remission.
2. Treatment of MCN with a reduced dose of prednisolone in combination with active vitamin D reduces the incidence of both the observed and subjective adverse events compared with the standard dose of prednisolone.
3. Patients with MCN and a fast turnover of prednisolone have a lower rate of remission, longer time to remission, and fewer adverse events than patients with a slow turnover of prednisolone.

### Trial design {8}

The trial design is a non-inferior, open-label, randomised, controlled multicentre study including 96 patients with primary, biopsy-proven MCN followed at renal departments in Denmark. The patients are randomised to the following:

- Standard prednisolone (1 mg/kg/day, maximum 80 mg/day), or
- Reduced prednisolone (0.5 mg/kg/day) and active vitamin D (alfacalcidol 0.5 μg/day).

When remission is achieved, prednisolone will be taped slowly according to an individual plan (defined by body weight at baseline and the result of randomisation). In both groups, prednisolone will be discontinued 25 weeks after remission. Alfacalcidol will be discontinued at the same time. The patients are followed until first relapse of MCN or for 1 year after remission.

## Methods: participants, interventions, and outcomes

### Study setting {9}

Patients in the trial will be recruited and followed from the following Danish study sites:

Department of Nephrology, University Hospital Aarhus

Department of Nephrology, University Hospital Copenhagen, Rigshospitalet

Department of Nephrology, University Hospital in Odense

Department of Nephrology, University Hospital in Aalborg

Department of Internal medicine, Renal unit, Regional Hospital Viborg

Department of Internal medicine, Renal unit, Regional Hospital Unit West Jutland, Holstebro

Department of Internal medicine, Renal unit, Hospital of Lillebaelt, Kolding

Department of Internal medicine, Renal unit, Sydvestjysk hospital, Esbjerg

Department of Internal medicine, Renal unit, University Hospital of Zealand, Roskilde

Department of Internal medicine, Renal unit, Nordsjællands Hospital, Hillerød

Department of Nephrology, Herlev and Gentofte Hospital, Herlev

Each site has a designated local investigator who will be responsible for obtaining informed consent, inclusion, and follow-up in the trial, including registry of data in the study database.

Furthermore, the trial has a coordinating investigator, who in cooperation with the sponsor is responsible for the trial, and a member of the ADAPT Trial Management Group.

### Eligibility criteria {10}

Inclusion criteria:

1. MCN verified by kidney biopsy
2. Nephrotic syndrome at diagnosis (defined as albuminuria > 3 g/day and hypoalbuminemia by local reference range)
3. Age ≥ 18 years

Exclusion criteria:

1. Secondary MCN due to, e.g. systemic immune-mediated disease, lymphoma, solid cancer, etc., based on the investigator’s assessment.
2. An episode of nephrotic syndrome within 5 years from inclusion and/or previous treatment with immunosuppressive medication other than prednisolone
3. Active cancer disease, except from basal cell carcinoma
4. Pregnancy
5. eGFR < 30 ml/min/1,73 m^2^ (CKD-EPI)
6. Unable to receive information and/or provide informed consent
7. Allergy or intolerance to prednisolone or alfacalcidol

### Who will take informed consent? {26a}

The local investigator (MD) will provide oral and written information about the study to all eligible patients and ask for consent to participate in the study. If patients agree to participate, they will be required to sign written informed consent.

### Additional consent provisions for collection and use of participant data and biological specimens {26b}

Not applicable.

### Interventions

#### Explanation for the choice of comparators {6b}

The choice of comparator is based on international guidelines (prednisolone 1 mg/kg/day) [8].

#### Intervention description {11a}

Patients will be randomised to either standard treatment with prednisolone 1 mg/kg/day or treatment with prednisolone 0.5 mg/kg/day combined with alfacalcidol 0.5 μg/day. The starting dose will remain unchanged until remission of MCN. After remission, prednisolone will be tapered slowly and discontinued after 25 weeks. All trial sites follow the same tapering regimen. The alfacalcidol dose remains unchanged while the patients receive prednisolone and is discontinued along with prednisolone.

#### Criteria for discontinuing or modifying allocated interventions {11b}

Prednisolone dose in both arms as well as alfacalcidol dose in the experimental arm may be reduced or discontinued if patients develop severe adverse events to these drugs as assessed by the local investigator. If possible, any deviation from the dosing regimen should be discussed with the sponsor prior to modification. Examples of severe adverse events are hypercalcaemia due to alfacalcidol treatment or psychoses, osteoporotic fracture, development of diabetes, or severe infections during high-dose prednisolone treatment.

#### Strategies to improve adherence to interventions {11c}

Prednisolone and alfacalcidol will be provided free of charge to patients from the local renal department. At each visit, the patients will be asked to bring study medication to be counted by the study nurse and registered in an individual medication log to ensure adherence to the medication. If the patients absent from study visits or biological tests, the study nurse will contact them by phone and rearrange visits to ensure adherence to the study protocol.

#### Relevant concomitant care permitted or prohibited during the trial {11d}

During the trial, patients may receive additional treatment for hypertension, hyperlipidaemia, and fluid control as well as prophylaxis targeting osteoporosis, gastric ulcers, and thrombosis according to local guidelines. The patients cannot receive other immunosuppressants or active vitamin D during the trial.

### Provisions for post-trial care {30}

Trial participants are covered by the national standard patient insurance. In case of harms related to the trial participation, patients will be compensated according to the national established guidelines. The local renal departments will be responsible for the post-trial care according to local procedures. Participants in the trial will post-trial be followed at their local nephrologists at the renal departments as traditionally.

### Outcomes {12}

Remission is defined by a urine-protein/creatinine ratio < 0.4 g/g and/or a urine-albumin/creatinine ratio < 0.3 g/g on two consecutive tests.

Relapse is defined as the recurrence of proteinuria, i.e. a urine-protein/creatinine ratio > 0.75 g/g and/or urine-albumin/creatinine ratio > 0.5 g/g in two consecutive tests and a decline in p-albumin of > 3 g/l.

Primary endpoints:

1. Rate of remissions after 16 weeks.
2. Time from diagnosis to remission.

Secondary endpoints:

1. Rate of relapse within 1 year after remission.
2. Time from remission to relapse.
3. Rate of adverse events and number of hospital admissions.
4. The associations between genetic variations in liver enzyme genes, the concentration of prednisolone in saliva, and remission or relapse.

Safety endpoints:

1. Patient survival
2. Acute kidney injury (AKI stage 2 or 3 as defined by KDIGO [31])
3. Serious infections requiring admission
4. Persistent hypercalcaemia (> 2 weeks) defined by an elevated plasma-ionised-calcium and not normalising by reducing alfacalcidol dose.
5. Severe adverse effects to prednisolone, e.g. gastrointestinal bleeding and severe depression

### Participant timeline {13}

See Fig. 1.

**Fig. 1:**
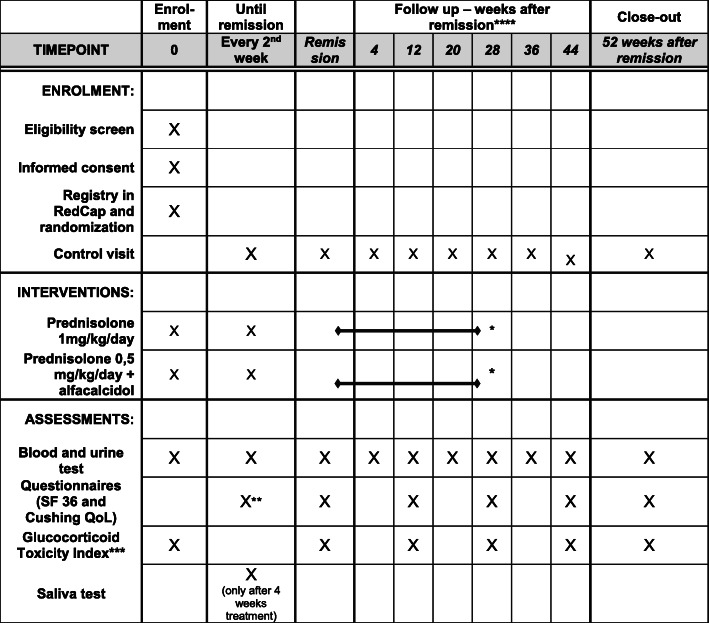
Participant timeline

### Sample size {14}

Based on current data, 80% of patients are expected to achieve remission after 16 weeks of standard treatment [8]. It is considered clinically significant if the remission rate in the low prednisolone + alfacalcidol regimen is less than 90% of the standard treatment regime. Using a non-inferior design with 80% power and a P value of = 0.05, we estimate that 43 patients in each group would need to complete the study. To allow for withdrawals, the study will recruit 96 patients. The incidence of adult MCN in Denmark is 7.3/m/year [4], and with all Danish renal departments participating in the trial, the inclusion is expected to be completed within 4 to 5 years.

### Recruitment {15}

Patients will be recruited from renal departments in Denmark by local investigators. To ensure that all incident patients diagnosed with MCN are considered to the study, agreements have been made with the local Pathology Departments to inform the coordinating investigator regularly about new renal biopsies suggesting MCN, allowing this to contact the relevant study site to make sure that all potential participants have been approached.

The first patient in the trial was included in September 2018 with more sites added during 2019 and 2020. The number of included patients is monitored continuously after initiation of the study and targeted efforts will be made to increase recruitment at participating study sites. This will include local analyses to identify any potentially eligible patients not being approached or informed about the study. During the study period, the coordinating investigator will be in personal contact with the local investigators. Study newsletters are regularly distributed to maintain awareness on the trial, and measures will be initiated to minimise any potential, practical obstacles preventing inclusion. If the inclusion of patients in the trial remains insufficient, the number of recruiting sites will be increased by including other renal departments in Scandinavia. Preliminary contact to the Renal Department at Akerhus University Hospital, Oslo, Norway, has been established.

### Assignment of interventions: allocation

#### Sequence generation {16a}

Patients are allocated to the treatment groups at a 1:1 ratio using permuted-block randomisation with random varying block sizes of 4, 6, and 8 stratified by age > 50 years (yes/no). The random allocation list is generated and uploaded to Research Electronic Data Capture (RedCap) by an independent service provider (Clinical Trial Unit, Dept. of Clinical Medicine, Aarhus University) maintaining proper concealment of randomisation.

#### Concealment mechanism {16b}

Randomisation is performed electronically by the local investigator using RedCap.

#### Implementation {16c}

The coordinating investigator and the Good Clinical Practice Unit will visit all trial sites before initiation of the trial to make sure that the local investigators and staff are informed about the protocol, the implementation, and the data management system. The RedCap data management system will be demonstrated, and the relevant access will be provided.

The allocation sequence provided by a RedCap service provider is blinded to all others, but the results of the randomisation are unblinded to both patients and study conductors. After being registered in RedCap at inclusion by the local investigators, the patients are allocated to the intervention according to randomisation.

### Assignment of interventions: blinding

#### Who will be blinded {17a}

The study is an open-label study, and neither the patients nor the investigators will be blinded to the interventions. Baseline data are collected independent of randomisation as it is collected prior to this.

#### Procedure for unblinding if needed {17b}

Not applicable.

### Data collection and management

#### Plans for assessment and collection of outcomes {18a}

The primary outcomes as well as some secondary outcomes are based on blood and urine tests sampled throughout the study period and analysed by using automated and standardised assays by the local Department of Clinical Biochemistry. Patient experienced adverse events are assessed using online questionnaires (SF36 and Cushing QoL) at weeks 2, 6, 10, and 14 before remission, at remission, and at weeks 12, 28, 44, and 52 after remission. Observer assessed, prednisolone-related morbidity is recorded using The Glucocorticoid Toxicity Index [25] reported every 3 months during the follow-up period.

Blood for analyses of genetic variations in the liver enzymes (CYP3A4, CYP3A5, ABCB1, and NR112) will be collected at baseline and stored in a biobank. Unstimulated saliva is sampled after approximately 4 weeks of prednisolone and before dose tapering using a home test (Salivette®). Saliva is sampled five times (0, 2, 6, 12, and 24 h) after taking oral prednisolone. Saliva samples will be stored in the biobank and analysed by liquid chromatography tandem mass spectrometry method in the end of the trial. Concentrations of prednisolone in saliva will be compared to the rate of and time to remission as well as the relapse rate and incidence of adverse events.

#### Plans to promote participant retention and complete follow-up {18b}

Data collected from participants who deviate from the intervention protocol or discontinue from the trial will be analysed and included in data management when relevant unless participant refuses.

### Data management {19}

Data will be collected in an electronic case report file (eCRF) management system RedCap and managed in accordance with regulatory requirements. Only members of the study team (investigators and study nurses) will have access to patient data. The investigators and study nurses are responsible for maintaining the data available for inspection by the regulatory authorities at any time.

### Confidentiality {27}

Personal information about enrolled participants will be stored in the secure RedCap platform, in accordance with authorities’ regulations and General Data Protection Regulation (GDPR). Data potentially shared will be anonymised. Saliva tests and gene samples will be anonymised before analysed by external laboratories.

### Plans for collection, laboratory evaluation, and storage of biological specimens for genetic or molecular analysis in this trial/future use {33}

Standard biochemical analyses (blood and urine) will be performed in the local Department of Clinical Biochemistry using standardised assays with nationally standardised reference intervals. Biological material for research (blood and urine) will be collected, handled and stored at – 80 °C until analysis. Saliva test will be performed by the participants at home and returned by post to The Renal Research Department in Aarhus and stored at − 80 °C until analysed.

### Statistical methods

#### Statistical methods for primary and secondary outcomes {20a}

The primary endpoints are analysed as intention-to-treat. Since this is an open-label study, there is a risk of cross-over between the two intervention groups, but we expect high adherence to intervention groups. Anyway, additional treated-as analyses will be included. Secondary endpoints are analysed as differences in absolute and relative proportions. Factors affecting the primary endpoint (age, baseline eGFR, albuminuria, prednisolone dose or alternative saliva profile) will be identified using a generalised, linear model.

#### Interim analyses {21b}

No interim analyses are planned.

#### Methods for additional analyses (e.g. subgroup analyses) {20b}

Not applicable.

#### Methods in analysis to handle protocol non-adherence and any statistical methods to handle missing data {20c}

Missing data will be resolved by carry-over of the last set of recorded data.

#### Plans to give access to the full protocol, participant level-data, and statistical code {31c}

Access to the datasets used and analysed during the study are available in a fully anonymised form from the sponsor on reasonable request.

### Oversight and monitoring

#### Composition of the coordinating centre and trial steering committee {5d}

The ADAPT Trial Management Group consists of the sponsor and the coordinating investigator. They are responsible to ensure that collaborative sites adhere to the study protocol, the appropriate contact to authorities, and appropriate data management and analyses. The ADAPT Trial Management Group will be available by e-mail or phone in case of urgent problems or concerns relating to the trial.

As a part of networking, investigators and collaborating colleagues will be invited for a yearly, half-day seminar to receive an update on the trial and to discuss relevant new research in nephrotic syndrome and MCN.

#### Composition of the data monitoring committee, its role and reporting structure {21a}

The trial has no attached Data Management Committee. The ADAPT Trial Management Group will supervise and monitor data collection and the reporting structure in corporation with the authorities (e.g. Good Clinical Practice (GCP) units). The potential benefits from reducing cumulative prednisolone dose are believed to override the risk of failure to achieve remission in the lower prednisolone dose regimen. Data uploaded in eCRF (RedCap) will continuously be monitored by the ADAPT trial Management Group and by the Good Clinical Practice (GCP) units in Denmark during the trial period.

### Adverse event reporting and harms {22}

Subjective events during the trial will be reported by patients in questionnaires (SF36 and Cushing QoL). Local investigators will report adverse events after results of blood test (development of diabetes, infections, hyperlipidaemia), examinations (hypertension, weight gain, skin toxicity), and questionnaires (glucocorticoid toxicity index). All the events will be collected and stored in RedCap. In case of severe adverse events, the sponsor will be informed and report it to relevant authorities.

### Frequency and plans for auditing trial conduct {23}

The ADAPT Trial Management Group will continuously be auditing the trial conduct by conversations and meetings with the local investigators and by monitoring data reporting in RedCap. During the trial period, the coordinating investigator will be in close contact with all the sites, and in case of difficulties of any kind, the coordinating investigator will contact the relevant site investigator to resolve the issue to make sure of optimal trial conduct.

### Plans for communicating important protocol amendments to relevant parties (e.g. trial participants, ethical committees) {25}

In case of protocol modifications, all relevant parts will be informed by e-mail or phone.

### Dissemination plans {31a}

The results of the trial, positive as well as negative, will be submitted to peer reviewed, international journals and presented at conferences and meetings. The participants will be informed on the patients’ association “Nyreforeningens” website (www.nyre.dk).

## Discussion

Lower dose of prednisolone in combination with active vitamin D in MCN might be an applicable option for sufficient disease control as a few small randomised studies suggest [14, 32–34].

Patients with MCN and nephrotic syndrome often have no co-morbidities. When being diagnosed with MCN, they experience symptoms such as oedema, fatigue, and foaming urine. After initiating medical treatment, they often experience additional changes and events, which can be related to both the disease and the treatment strategy. Is it imperative that we trace a treatment strategy that is minimising treatment-related adverse events.

This trial uses a predefined electronic randomisation open-labelled design. The open-label design reflects the daily clinically reality. Adherence to treatment strategy and patient compliance will be monitored, e.g. by counting pills. It is commonly anticipated that lower dose of prednisolone decreases adverse events. This might encourage a speedy reduction of prednisolone dose or cross-over from standard to reduced dose of prednisolone especially if the patient prior to treatment has obstacles with e.g. obesity, osteoporosis, hypertension or other known adverse events to prednisolone treatment. Local investigators will be encouraged to adhere to treatment strategy and change in prednisolone dose can only be done after agreement with sponsor or his substitute. The trial will provide evidence to the commonly anticipated belief that the number of adverse events increases with the dose of prednisolone. Alfacalcidol is a well-known treatment among nephrologists and widely used in other patients with chronic nephropathies. Using it in patients with MCN is newfound but expected to be tolerable.

Self-reported, prednisolone associated adverse events have not been systematically evaluated in MCN, but in renal transplant patients, the cumulative dose of prednisolone correlates with reported adverse effects using the Cushing QoL questionnaire [26, 27] and SF36 [35].

This trial should allow us to determine if low dose prednisolone in combination with active vitamin D is clinically as efficient as current standard therapy to induce remission in MCN with the potential to reduce significant adverse events. This may lead to changes of current guidelines for the treatment of MCN. If so, the results potentially can inspire to additional trials in other prednisolone-treated diseases to examine reduced dose of prednisolone.

Furthermore, the trial should provide additional information on the ability of a saliva test to characterise prednisolone pharmacokinetics in individuals and the ability of this to predict the prednisolone dose-dependent treatment response. In the future, such tests may allow for a more personalised treatment and dosing regimen not only in patients with MCN but also in other patients requiring prednisolone treatment.

## Trial status

Currently version of protocol: Version 10.01.2020

Recruitment began in May 2018 and is expected to be completed by 2023.

## Data Availability

Not applicable.

## Abbreviations

ADAPT: (name of the trial) Activated vitamin D and prednisolone treatment
MCN: Minimal change nephropathy
eGFR: Estimated glomerular filtration rate
KDIGO: Kidney Disease: Improving Global Outcomes
QoL: Quality of life
SF 36: Short form 36
CKD-EPI: Chronic Kidney Disease Epidemiology Collaboration
eCRF: Electronic case report file
RedCap: Research Electronic Data Capture

## References

[1] Hogan J, Radhakrishnan J (2013). The treatment of minimal change disease in adults. J Am Soc Nephrol..

[2] Vivarelli M, Massella L, Ruggiero B, Emma F (2017). Minimal change disease. Clin J Am Soc Nephrol..

[3] Vaziri ND (2016). Disorders of lipid metabolism in nephrotic syndrome: mechanisms and consequences. Kidney Int.

[4] Heaf J, Løkkegaard H, Larsen S (1999). The epidemiology and prognosis of glomerulonephritis in Denmark 1985-1997. Nephrol Dial Transplant..

[5] Seeger H, Fehr T (2016). Nephrotic syndrome in adult patients--etiology and complications. Praxis (Bern 1994).

[6] Ordoñez JD, Hiatt RA, Killebrew EJ, Fireman BH (1993). The increased risk of coronary heart disease associated with nephrotic syndrome. Kidney Int..

[7] Suri D, Ahluwalia J, Saxena AK, Sodhi KS, Singh P, Mittal BR, et al. Thromboembolic complications in childhood nephrotic syndrome: a clinical profile. Clin Exp Nephrol. 2014;18:803–13. 10.1007/s10157-013-0917-2 24346593

[8] Kidney Disease Improving Global Outcomes (2012). KDIGO Clinical practice guideline for glomerulonephritis. Kidnet Int Suppl..

[9] Raja K, Parikh A, Webb H, Hothi D (2017). Use of a low-dose prednisolone regimen to treat a relapse of steroid-sensitive nephrotic syndrome in children. Pediatr Nephrol..

[10] Waldman M, Crew RJ, Valeri A, Busch J, Stokes B, Markowitz G, D'Agati V, Appel G (2007). Adult minimal-change disease: clinical characteristics, treatment, and outcomes. Clin J Am Soc Nephrol..

[11] Shinzawa M, Yamamoto R, Nagasawa Y, Oseto S, Mori D, Tomida K, Hayashi T, Izumi M, Fukunaga M, Yamauchi A, Tsubakihara Y, Isaka Y (2014). Comparison of methylprednisolone plus prednisolone with prednisolone alone as initial treatment in adult-onset minimal change disease: a retrospective cohort study. Clin J Am Soc Nephrol..

[12] Lee H, Yoo KD, Oh YK, Kim DK, Oh K-H, Joo KW, Kim YS, Ahn C, Han JS, Lim CS (2016). Predictors of relapse in adult-onset nephrotic minimal change disease. Medicine (Baltimore)..

[13] Black DA, Rose G, Brewer DB (1970). Controlled trial of prednisone in adult patients with the nephrotic syndrome. Br Med J..

[14] Imbasciati E, Gusmano R, Edefonti A, Zucchelli P, Pozzi C, Grassi C, Della Volpe M, Perfumo F, Petrone P, Picca M (1985). Controlled trial of methylprednisolone pulses and low dose oral prednisone for the minimal change nephrotic syndrome. Br Med J..

[15] Zhao L, Cheng J, Zhou J, Wu C, Chen J (2015). Enhanced steroid therapy in adult minimal change nephrotic syndrome: a systematic review and meta-analysis. Intern Med..

[16] Liu LJ, Lv JC, Shi SF, Chen YQ, Zhang H, Wang HY (2012). Oral calcitriol for reduction of proteinuria in patients with IgA nephropathy: a randomized controlled trial. Am J Kidney Dis..

[17] Fishbane S, Chittineni H, Packman M, Dutka P, Ali N, Durie N (2009). Oral paricalcitol in the treatment of patients with CKD and proteinuria: a randomized trial. Am J Kidney Dis..

[18] Szeto CC, Chow KM, Kwan BCH, Chung KY, Leung CB, Li PKT (2008). Oral calcitriol for the treatment of persistent proteinuria in immunoglobulin a nephropathy: an uncontrolled trial. Am J Kidney Dis..

[19] Mirković K, van den Born J, Navis G, de Borst MH (2011). Vitamin D in chronic kidney disease: new potential for intervention. Curr Drug Targets..

[20] Agarwal R, Acharya M, Tian J, Hippensteel RL, Melnick JZ, Qiu P (2005). Antiproteinuric effect of oral paricalcitol in chronic kidney disease. Kidney Int..

[21] Deng J, Zheng X, Xie H, Chen L (2017). Calcitriol in the treatment of IgA nephropathy with non-nephrotic range proteinuria: a metaanalysis of randomized controlled trials. Clin Nephrol..

[22] Barragry JM, Carter ND, Beer M, Cohen RD, France MW, Auton JA, Boucher BJ (1977). Vitamin-D metabolism in nephrotic syndrome. Lancet..

[23] Li X-H, Huang X-P, Pan L, Wang C-Y, Qin J, Nong F-W, Luo YZ, Wu Y, Huang YM, Peng X, Yang ZH, Liao YH (2016). Vitamin D deficiency may predict a poorer outcome of IgA nephropathy. BMC Nephrol..

[24] Bergmann TK, Barraclough KA, Lee KJ, Staatz CE (2012). Clinical pharmacokinetics and pharmacodynamics of prednisolone and prednisone in solid organ transplantation. Clin Pharmacokinetics.

[25] Miloslavsky EM, Naden RP, Bijlsma JWJ, Brogan PA, Brown ES, Brunetta P, et al. Development of a Glucocorticoid Toxicity Index (GTI) using multicriteria decision analysis. Ann Rheum Dis. 2017;76(3):543–6. 10.1136/annrheumdis-2016-210002 27474764

[26] Webb SM, Badia X, Barahona MJ (2008). Colao a, Strasburger CJ, Tabarin a, et al. Evaluation of health-related quality of life in patients with Cushing’s syndrome with a new questionnaire. Eur J Endocrinol..

[27] Nelson LM, Forsythe A, McLeod L, Pulgar S, Maldonado M, Coles T, Zhang Y, Webb SM, Badia X (2013). Psychometric evaluation of the Cushing’s quality-of-life questionnaire. Patient..

[28] Wight JP, Edwards L, Brazier J, Walters S, Payne JN, Brown CB (1998). The SF36 as an outcome measure of services for end stage renal failure. Qual Heal Care..

[29] Teeninga N, Guan Z, Freijer J, Ruiter AFC, Ackermans MT, Kist-van Holthe JE (2013). Monitoring prednisolone and prednisone in saliva: a population pharmacokinetic approach in healthy volunteers. Ther Drug Monit..

[30] Teeninga N, Guan Z, Stevens J, Kist-van Holthe JE, Ackermans MT, van der Heijden AJ, et al. Population pharmacokinetics of prednisolone in relation to clinical outcome in children with nephrotic syndrome. Ther Drug Monit. 2016. 10.1097/FTD.0000000000000308 27120177

[31] Kidney Disease: Improving Global Outcomes (KDIGO) Acute Kidney Injury Work Group (2012). KDIGO clinical practice guideline for acute kidney injury. Kidney Int Suppl.

[32] Kim YC, Lee TW, Lee H, Koo HS, Oh KH, Joo KW, Kim S, Chin HJ (2012). Complete remission induced by tacrolimus and low-dose prednisolone in adult minimal change nephrotic syndrome: a pilot study. Kidney Res Clin Pract..

[33] Li X, Liu Z, Wang L, Wang R, Ding G, Shi W, Fu P, He Y, Cheng G, Wu S, Chen B, du J, Ye Z, Tao Y, Huo B, Li H, Chen J (2017). Tacrolimus monotherapy after intravenous methylprednisolone in adults with minimal change nephrotic syndrome. J Am Soc Nephrol..

[34] Gulati A, Sinha A, Sreenivas V, Math A, Hari P, Bagga A (2011). Daily corticosteroids reduce infection-associated relapses in frequently relapsing nephrotic syndrome: a randomized controlled trial. Clin J Am Soc Nephrol..

[35] Bergmann TK, Isbel NM, Ostini R, Barraclough KA, Campbell SB, McWhinney BC (2015). Exploratory study of total and free prednisolone plasma exposure and cushingoid appearance, quality of life and biochemical toxicity in adult male kidney transplant recipients. Clin Drug Investig..

